# Correlation of Pleural and Pulmonary Ultrasound with the Severity of Autoimmune Interstitial Lung Disease

**DOI:** 10.5152/ArchRheumatol.2025.10999

**Published:** 2025-09-01

**Authors:** Luis Javier Cajas Santana, Satiago Cuero, Gabriela Guerrero, Mayelin Ceballos, María Carolina Torres Villarreal

**Affiliations:** 1 Department of Rheumatology, Hospital Universitario Nacional de Colombia, Bogotá, Colombia; 2 Department of Rheumatology, Universidad Nacional de Colombia, Bogotá, Colombia; 3 Department of Internal Medicine, Universidad Nacional de Colombia, Bogotá, Colombia; 4 Department of Pneumology, Hospital Universitario Nacional de Colombia, Bogotá, Colombia

**Keywords:** Interstitial lung disease, rheumatoid arthritis, systemic sclerosis, ultrasonography

## Abstract

**Background/Aims:**

Interstitial lung disease is one of the most important complications in autoimmune diseases. The extent of involvement in tomography is crucial for therapeutic decision-making. Pleuropulmonary ultrasound is helpful in screening and correlates with severity. Using the Goh method, the objective is to analyze the correlation between ultrasound findings and the quantified extent on high-resolution computed tomography (HRCT).

**Materials and Methods:**

The HRCT images of patients with rheumatoid arthritis or systemic sclerosis were analyzed using the Goh method. The data were compared with the number of B-lines and ultrasound pleural abnormalities. The correlation was determined using Spearman’s Rho statistic, and receiver operating characteristic curve analysis was performed. The sensitivity, specificity, and cutoff points were calculated for each ultrasound finding to detect severe disease.

**Results:**

A total of 71 patients were included. Almost half of the patients (56%) were involved in less than 5% of extent in HRCT; an average disease extent was 11% for the whole population. The correlation (Rho) between the extent and the total B-line count was 0.58 and 0.61 (*P* < .001), and for pleural abnormalities, 0.60 and 0.59 (*P* < .001) in linear and convex images, correspondingly. The areas under the curve were high for both ultrasound abnormalities and in both forms of images, consistently exceeding 0.7. Regarding the cutoff values, a number greater than 20 B-lines has a specificity close to 90% for detecting extensive disease, as well as 7 or more pleural abnormalities.

**Conclusion:**

The count of B-lines and the number of pleural abnormalities on lung ultrasound correlate well with the extent of the disease and can help determine its severity.

**Main Points**

- There is a significant correlation between the B-line count or pleural abnormalities and disease severity on HRCT as assessed by the Goh method.
- No significant differences were found between linear and convex images regarding the association between ultrasound findings and disease extent on HRCT.
- A number greater than 20 B-lines or seven pleural abnormalities has a specificity close to 90% for detecting extensive disease.

## Introduction

Interstitial lung disease (ILD) is one of the most significant complications in various autoimmune diseases, leading to increased morbidity and mortality.^1,2^ Within the proposed algorithms and recommendations for diagnosing and treating autoimmune-related ILD (AI-ILD), quantifying the extent is crucial for classifying patients with severe or poor prognosis.^3,4^ This determination has been extensively studied in systemic sclerosis (SSc) patients and extrapolated to other conditions, primarily rheumatoid arthritis (RA). In this first group of patients, interstitial involvement with an extent greater than 20%, categorized as severe disease, has been identified as a determinant of mortality and greater complications, making it a criterion for initiating treatment.^5^

Several methods exist to determine the extent, but the most commonly used and widespread method has been quantification by the Goh method, which classifies patients as having severe disease or not, analyzing 5 slices of high-resolution computed tomography (HRCT).^5,6^ This method has not only been used in clinical trials and demonstrated its utility in essential outcomes, unlike others, making it the one with the most significant validity, but it has also been employed to assess patient evolution and changes from the initiation of treatments, defining failure or disease progression,^7^ and serving as a criterion to determine if a progressive fibrosing variant is present.^8^ This has been predominantly used and validated in patients with SSc; however, it has also been utilized in RA to assess the disease and monitor treatment response in its progression.^9,10^

Despite this, the use of HRCT has limitations, especially when frequent monitoring is required, such as the time it takes for a patient with this severe complication to return for a follow-up appointment with images, recognizing that a significant number of patients progress rapidly and lose pulmonary functional capacity in a few months, the radiation doses required with frequent HRCT, and the potential increased costs to the healthcare system.

An alternative is the use of pleuropulmonary ultrasound in the evaluation of AI-ILD. Several studies have demonstrated high sensitivity (greater than 90%) in detecting this complication compared to HRCT, with specificity ranging between 80 and 85% in most reports.^11-15^

Recently, it has been used in patients primarily with SSc and RA, mostly yielding excellent results for screening and early detection due to its high sensitivity in relation to a high number of B-lines, with different cutoff points (ranging from 10 to 15) depending on the protocol used, whether it’s a comprehensive one with over 50 views or a shortened one with 14, 10, or even fewer and, to a lesser extent, pleural abnormalities.^16,17^

In addition to the finding of B-lines, more recently, it was found that pleural irregularities are significantly related to the presence of ILD. In 2012, Fuerst et al^18^ found that no patient without ILD-SSc presented abnormal pleural findings. The same author in 2014 confirmed this finding in patients with RA,^19^ where all those with abnormalities on tomography compatible with ILD presented pleural abnormalities defined as continuity alterations or granulations. Despite this, so far, despite the recognition of pleural abnormalities as a marker of disease, their quantification of the extent of the disease has yet to be studied.

Ultrasound is a low-cost, easy-to-perform, and analyze tool that requires short training. It can be done at the bedside and is rapid, taking approximately 8 minutes.

To date, ultrasound findings have been studied for the presence or absence of disease and the extent of involvement. Most studies have used scales different from Goh’s, excluding pleural findings. Therefore, this study aims to analyze the relationship between pleural and pulmonary findings and the extent of the disease measured by Goh using linear and convex images.

## Methods

Patients who had attended the rheumatology clinic of a university hospital for over 1 year were included in the study. These patients had undergone HRCT in the previous 3 months for the study of ILD associated with RA, diagnosed according to the 2010 ACR/EULAR criteria, or SSc, according to the 2013 ACR/EULAR criteria. Patients with exacerbation or worsening of symptoms after the HRCT were excluded. Those with other potential causes of ILD, poor-quality HRCT that did not allow proper assessment, or HRCT taken more than 3 months apart from clinical and ultrasound evaluation were also excluded.

The present study meets the requirements stipulated by the Ministry of Health of Colombia and has been approved by an ethics committee of National University of Colombia (January 21, 2022. HUN. CEI-2021-08-04). All patients signed informed consent to be included in the study.

### Tomography Reading

High-resolution computed tomography images meeting sufficient technical requirements were obtained. The 5 slices necessary for quantifying disease extent described by Goh were extracted:^5,6^ 1. Exit of the great vessels, 2. Carina level, 3. Confluence of pulmonary veins, 4. Halfway between 3 and 5, and 5. Immediately superior to the right hemidiaphragm. The lesions to be quantified were interstitial alterations classified as reticulation, traction bronchiectasis, honeycombing, and ground-glass opacities. Two physicians with training and experience in ILD anonymously read the topographies. They were blinded to the clinical history and the ultrasound findings.

In case of significant discordance, a third opinion was sought. The average extent of the 2 readers was taken, and in case of a discrepancy greater than 5%, the third opinion was considered. An initial pilot test was conducted to clarify the method used.

### Pulmonary Ultrasound Study

A physician experienced in pulmonary ultrasound, blinded to patient data and HRCT, took static images^13^ according to a shortened protocol of 14 points described in the literature, including images taken from both linear and virtual convex modalities. The convex images were taken from a linear transducer, with the possibility, due to the ultrasound machine’s features, of converting it into a convex one depending on the ultrasound emission mode.

A frequency of 8-10 MHz was used, with gain between 40 and 60 and depth as needed. The window was taken parallel to the intercostal space. The equipment used was a GE Logiq E with a linear transducer of 6-13 MHz. Three rheumatologists with prior training in pulmonary ultrasound evaluated the images.

Each observer performed a B-line count on all static images and determined the number of windows with pleural abnormalities associated with ILD, with a maximum of 14 points. A pleural abnormality is defined as irregularity, fragmentation, presence of subpleural nodules, or loss of continuity in the pleural line. An initial pilot test was conducted to clarify the method used.

### Objectives

The main objective was to determine the correlation between the extent of ILD using the Goh method and ultrasound findings, both B-line count and the number of windows with pleural abnormalities for both images (linear and convex virtual conversion). Secondary objectives were to determine the performance of ultrasound for the detection of abnormalities at different severity points (5%, 10%, or 20% of extent in HRCT), taken arbitrarily, considering that the method aims to approximate the extent of the disease in multiples of 5 and that in patients with RA, it has been found that 10% might already be considered a severe disease;^20^ and to determine sensitivity and specificity values for different cutoff points.

### Statistical Analysis

Descriptive statistics were used for qualitative variables, presenting means with standard deviations (SD) or medians with interquartile range, depending on the normality of the distribution. Categorical variables are described with relative and absolute frequencies. The disease extent variable was analyzed as a continuous variable and categorized as severe or not severe based on whether it was greater than or less than 20% or 10%. The correlation between continuous variables was determined using Spearman’s Rho statistic for the primary objective. The receiver operating characteristic (ROC) curve analysis was performed for the secondary purpose. Sensitivity, specificity, and correct classification percentage were estimated for each cutoff point. A statistically significant *P*-value was considered less than .05.

## Results

Initially, data from 86 patients were collected, but ultimately, 71 patients with adequate HRCT and pulmonary ultrasound images were included in the analysis. Table 1 presents the characteristics of the study population and a summary of HRCT and pulmonary ultrasound findings.

The majority of patients were female (82%), with 34 diagnosed with RA (47.2%) and 37 with SSc (52.8%). Only 25% of patients had a history of tobacco exposure. Most RA patients were seropositive; among SSc patients, the majority had anti-centromere antibodies (43.2%) or SCL-70 antibodies (20.6%).

Regarding HRCT evaluation, many patients showed less than 5% (56%) involvement, while the other half had varying degrees of moderate to severe involvement. The average extent of the disease in the studied population was 11%. The mean disease duration was 5 and 8 years for RA and SSc patients, respectively. Methotrexate was the most used treatment; one-third had steroids, and 20% had biological therapy in RA patients.

Cyclophosphamide was the most frequent treatment for patients with SSc because of lung involvement, and only one-fifth had mycophenolate.

**Figure 1.**
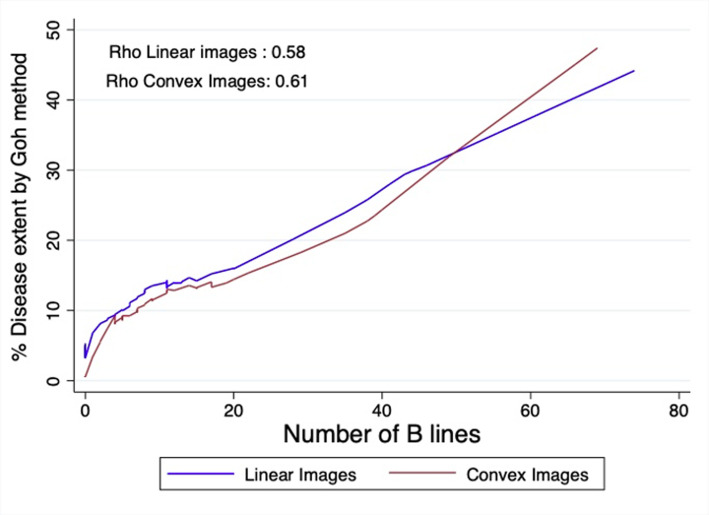
Relationship between the number of B-scan lines in linear and convex vision and the extent observed through tomography.

**Figure 2.**
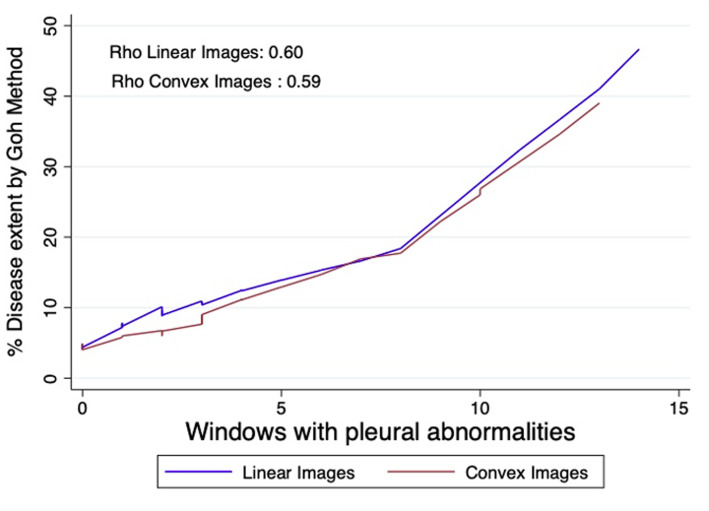
Relationship between the number of pleural abnormalities in linear and convex vision and the extent observed through tomography.

The correlation (Rho) between the extent of ILD measured by the Goh method and the total B-line count (Rho = 0.58 and 0.61 respectively) (*P* < .001) and for pleural abnormalities (Rho = 0.60 and 0.59 respectively) (*P* < .001) in linear and convex images are represented in Figures 1 and 2.

When the number of lines and windows with pleural irregularity was included, a slight increase in the correlation with disease extent was observed, primarily in linear images (Rho = 0.75, *P* < .001) and lower in convex images (Rho= 0.64, *P* < .001).

The ROC curve analysis was conducted to determine ultrasound performance in detecting abnormalities at different severity points (5%, 10%, or 20% of extent in HRCT) for linear and convex views for variables, B-line count, and pleural abnormalities. The results are presented in Table 2.

No significant differences were found between the 2 image modalities, whether linear or convex. The AUC for detecting disease over 10% is, on average, slightly higher than for 20%, although both values are above 0.7. The same performance was observed for both B-line determination and pleural abnormality assessment.

Tables 3 and 4 present sensitivity and specificity results for each cutoff point and extent greater than 20%.

## Discussion

Lung ultrasound is a method under study for ILD, primarily in SSc and RA. It is based on detecting abnormalities such as B-lines and pleural changes. Compared to the gold standard, HRCT, these abnormalities have shown high sensitivity, even in the early stages of the disease.^11-17^

Since the beginning of ultrasound use, it has been associated with the degree of disease severity. In 2004, Jambrick^21^ found an association between the number of B-lines and the degree of lung water on HRCT, and in 2009, Gargani^11^ found an association with the degree of fibrosis measured by the Warrick scale in patients with SSc (*r* = 0.72). The same finding was found years later in 2018 by Tardella^22^ (rho = 0.81).

This study represents one of the first attempts to conduct a correlation of pulmonary ultrasound study in patients with ILD about the extent found using the widely disseminated Goh method, uncovering a good correlation with both the B-line count and the number of pleural irregularities, both in linear and convex images without finding any significant differences, which are straightforward findings to visualize and may have a lower degree of subjectivity.

This finding is important because it would allow for a quicker assessment, even during the same consultation, to obtain data on disease severity, especially severe involvement considered as more than 20%, which, as mentioned, is part of the criteria for determining the initiation of treatment. This is demonstrated by an area under the curve for both B-lines and pleural abnormality with adequate values compared to the gold standard. Based on the study, the presence of more than 20 B-lines would have a high specificity close to 90% in detecting severe disease, whether in linear or convex views, as well as more than 7 windows with pleural abnormalities.

Conversely, a low B-line count of less than 5 or fewer than 2 abnormal pleural windows would have high sensitivity, close to 90%, which is crucial not only for general screening in all autoimmune patients at risk of ILD but also for quickly determining the possibility of it being severe and requiring immediate immunosuppressive treatment. Therefore, the combined evaluation of these 2 independent findings is superior to just the B-line count, providing more information in the same assessment without a significant increase in time, considering that several pulmonary alterations that can generate false positives in the line count would not give pleural abnormalities.

Similarly, yet to be studied, this correlation with the quantification of the extent may play a role in monitoring disease progression to determine the success or failure of treatments for progressive fibrosing variants.

As previously mentioned, these findings were found in previous studies, which confirms that ultrasound studies can also determine the severity of the disease, not only with the study of B lines but also with pleural abnormalities.

Another critical finding is that no significant differences were observed between linear and convex images. This is important considering that the previous literature has been heterogeneous regarding which of these 2 forms of image capture or processing is better for the lung. Therefore, there is no standardized or widely used protocol. In this case, it is emphasized that linear images, taken with the same transducer used for musculoskeletal ultrasound studies, have the same capability, meaning cost savings on additional equipment.

As part of the difficulties encountered, erroneous B-line counts may have occurred due to the use of static images instead of video capture. However, there is no agreement on the best method, recognizing that, considering the movement of B-lines during the respiratory cycle, video capture could make counting more difficult, mainly when multiple B-lines are found, as their movement could overlap. It is also essential that the images were captured parallel to the intercostal space, contrary to most studies, limiting the acoustic window due to the shadow cast by the ribs. However, it is essential to take advantage of the entire acoustic extent that the equipment allows, as there may be alterations beneath the bone shadow that may not be perceived. Complete spirometry data, particularly FEV_1_ values, were not available. These values are especially relevant considering the percentage of smokers in the cohort.

An additional limitation is the low representation of severe cases with an extent greater than 20%, which is lower than reported in other cohorts, both in RA (30%-50%) and SSc (30%-60%), which may introduce some divergence in the determination of the test’s performance.

Considering the type of study, ultrasound will always have the possibility of depending on the reader, which has been extensively studied. Nevertheless, it has also been found that the training time to detect abnormalities is short for the analysis of pulmonary ultrasound images, as the alterations are generally quite noticeable.

Finally, although the period between ultrasound and tomography was short, less than 3 months, in this pathology, there may be clinical pictures of acute deterioration or even acute improvement after the initiation of immunosuppressive treatments, which could change the results. There are currently no studies on sensitivity to change or ultrasound as a form of evaluating treatment success or failure. Additionally, the absence of studies on the usefulness of ultrasound in follow-up, the chance of false positives related to other pulmonary diseases, and its inability to classify exactly the pattern or classification of interstitial lung abnormalities are significant restrictions. These promise additional research on this diagnostic approach in these patients.

In conclusion, there is a good correlation between the B-line count, the number of abnormal pleural findings, and the extent of the disease measured by Goh, with no significant differences between images taken linearly or convexly.

## Figures and Tables

**Table 1.** Description of the Population

Variable	Result
Gender (female)	82%
Diagnosis Rheumatoid arthritis Systemic sclerosis	34 (47.2%) 37 (52.8%)
Age (SD), years	62.2 (10.4)
Smokers	25%
Antibodies Centromere SCL-70 Rheumatoid factor Anti-CCP	16/37 (43.2%) 7/37 (20.6%) 32/34 (94%) 28/34 (82.3%)
Forced vital capacity (% of predicted) (SD)	86 (16)
Extent of disease in HRCT Up to 5% 5%-10% 10%-20% More than 20% Average (SD)	56 12 11 14 11 (15.7)
Number of B lines Linear images (SD) Convex images (SD)	9.3 (12.4) 11 (13.8)
Pleural abnormalities (number of windows) Linear images (SD) Convex images (SD)	Alteraciones pleurales (de ventanas anormales) 3 (3.7) 3.6 (3.6)

Anti-CCP, anti-cyclic citrullinated peptide; HRCT, high-resolution computed tomography; SCL-70, anti-Scl-70 antibody; SD, Standard deviation.

**Table 2.** Area Under the Curve (AUC) Values According to Different Severity Points in HRCT

Extent	Variable	AUC	95% CI
More than 5%	B lines-linear images	0.79	0.69-0.89
More than 10%		0.79	0.67-0.91
More than 20%		0.74	0.57-0.90
More than 5%	Pleural abnormalities-linear images	0.8	0.7-0.9
More than 10%		0.79	0.69-0.91
More than 20%		0.74	0.57-0.90
More than 5%	B lines-convex images	0.78	0.67-0.88
More than 10%		0.81	0.70-0.91
More than 20%		0.74	0.60-0.89
More than 5%	Pleural abnormalities-convex images	0.79	0.69-0.89
More than 10%		0.81	0.69-0.92
More than 20%		0.76	0.61-0.92

**Table 3.** Sensitivity and Specificity Values of the Number of B-Scan Lines and Pleural Abnormalities in Linear Vision for the Determination of an Extent Greater than 20%

Cutoff Point	Sensitivity B Lines (%)	Specificity B Lines(%)	Sensitivity Pleural(%)	Specificity Pleural(%)
(≥0)	100	0.00	100.00	0.00
(≥1)	92.31	37.93	84.62	39.66
(≥2)	84.62	43.10	76.92	56.90
(≥3)	76.92	46.55	69.23	65.52
(≥4)	69.23	48.28	61.54	74.14
(≥5)	61.54	55.17	53.85	77.59
(≥6)	53.85	56.90	46.15	82.76
(≥7)	53.85	63.79	46.15	87.93
(≥8)	46.15	65.52	38.46	96.55
(≥9)	46.15	72.41		
(≥11)	38.46	72.41	38.46	98.28
(≥12)	38.46	79.31	23.08	98.28
(≥13)	38.46	82.76	23.08	100.00
(≥14)	38.46	86.21	7.69	100.00
(≥15)	38.46	87.93	0	
(≥17)	30.77	87.93		
(≥20)	30.77	89.66		
(≥28)	30.77	93.10		
(≥35)	30.77	94.83		
(≥38)	30.77	96.55		
(≥41)	23.08	96.55		
(≥43)	23.08	98.28		
(≥44)	23.08	100.00		
(≥46)	15.38	100.00		
(≥74)	7.69	100.00		
(>74)	0.00	100.00		

**Table 4.** Sensitivity and Specificity Values of the Number of B-Scan Lines and Pleural Abnormalities in Convex Vision for the Determination of an Extent Greater than 20%

Cutoff Point	Sensitivity B lines (%)	Specificity B lines (%)	Sensitivity Pleural (%)	Specificity Pleural (%)
(≥0)	100.00	0.00	100.00	0.00
(≥1)	100.00	22.41	92.31	34.48
(≥2)	100.00	29.31	84.62	44.83
(≥3)	100.00	34.48	76.92	50.00
(≥4)	92.31	36.21	76.92	60.34
(≥5)	84.62	44.83	76.92	74.14
(≥6)	76.92	53.45	69.23	79.31
(≥7)	76.92	56.90	46.15	84.48
(≥8)	76.92	65.52	30.77	91.38
(≥9)	61.54	65.52	30.77	94.83
(≥11)	53.85	68.97	30.77	96.55
(≥12)	53.85	75.86	23.08	98.28
(≥14)	53.85	77.59	0	100.00
(≥15)	46.15	77.59		
(≥17)	38.46	81.03		
(≥19)	38.46	86.21		
(≥20)	38.46	87.93		
(≥22)	38.46	89.66		
(≥29)	38.46	91.38		
(≥35)	38.46	94.83		
(≥38)	38.46	96.55		
(≥39)	30.77	96.55		
(≥40)	30.77	98.28		
(≥49)	23.08	98.28		
(≥51)	15.38	98.28		
(≥66)	15.38	100.00		

## Data Availability

The data that support the findings of this study are available on request from the corresponding author.
